# Improving Anti-Corrosion and Conductivity of NiTi Alloy Bipolar Plate Used for PEMFCs via Nb Alloying

**DOI:** 10.3390/molecules30173658

**Published:** 2025-09-08

**Authors:** Ziyang Niu, Yingping Li, Yuanyuan Li, Xiaofen Wang, Yumin Pan, Zhuo He, Guohong Zhang, Zhen Wang, Qiongyu Zhou

**Affiliations:** 1 The State Key Laboratory of Refractories and Metallurgy, Wuhan University of Science and Technology, Wuhan 430081, China; niuziyang@wust.edu.cn (Z.N.); liyingping@wust.edu.cn (Y.L.); 2 Analytical and Testing Center, Wuhan University of Science and Technology, Wuhan 430081, China; zhangguohong@wust.edu.cn (G.Z.); wangzhen@wust.edu.cn (Z.W.); 3 Key Laboratory of Green Surface Technology and Functional Coatings for Materials, China National Light Industry, Foshan University, Foshan 528000, China; wangxiaofen@shu.edu.cn (X.W.); 20230580130@stu.fosu.edu.cn (Y.P.); 20220580212@stu.fosu.edu.cn (Z.H.); 4 School of Materials Science and Engineering, Shanghai University, Shanghai 200444, China

**Keywords:** NiTiNb alloy, corrosion resistance, interfacial contact resistance, bipolar plate, PEMFC

## Abstract

NiTi alloy has emerged as a promising bipolar plate (BP) material for proton exchange membrane fuel cells (PEMFCs), combining Ti-like corrosion resistance with Ni-like electrical conductivity through its intermetallic characteristics. However, its performance faces greater challenges under aggressive operating conditions (70 °C, F^−^-containing acidic solution with air bubbling). This study demonstrates that Nb alloying effectively enhances NiTi while preserving its balanced properties. The developed NiTiNb alloy exhibits improved performance with 26% lower corrosion current density (*i*_c_) and 29% reduced interfacial contact resistance (ICR) compared to conventional NiTi, effectively overcoming the conventional corrosion–conductivity trade-off in metallic BPs. The alloy also shows superior electrochemical stability and microhardness relative to pure Ti and Ni. These enhancements stem from a unique dual-phase microstructure comprising a NiTi (B2) matrix with continuous β-Nb grain boundary networks. During operation, this structure enables in situ formation of protective TiO_2_-Nb_2_O_5_ films while maintaining conductive Nb/Nb_2_O_5_ pathways and metallic Ni domains. The findings establish Nb alloying as a viable optimization strategy for NiTi-based BP substrate in demanding PEMFC applications.

## 1. Introduction

Hydrogen energy has emerged as a pivotal clean energy solution to address the dual crises of fossil fuel depletion and escalating greenhouse gas emissions [1]. Among various hydrogen utilization technologies, fuel cells (FCs) have garnered significant attention for their ability to convert chemical energy from hydrogen directly into electricity with exceptional efficiency and zero greenhouse gas emissions [2]. Proton exchange membrane fuel cells (PEMFCs), in particular, are renowned for their high energy efficiency, near-zero emissions, and rapid cold-start capability, making them one of the most viable power sources for next-generation electric vehicles and portable devices [3,4]. Within PEMFC stacks, bipolar plates (BPs) serve as one of the most critical components, accounting for 60–80% of the stack weight and 20–30% of the total cost [5]. To perform their essential functions (such as gas distribution, current collection, membrane electrode support, and heat discharge), BPs must satisfy stringent requirements, including excellent corrosion resistance in acidic environments, low interfacial contact resistance, and high mechanical robustness [6,7].

BP materials, including graphite, composites, and metals, have been extensively investigated over the past three decades [8]. Graphite, the earliest BP material developed, displays advantages such as low density, superior electrical conductivity, and outstanding chemical inertness [9]. However, its inherent brittleness and poor manufacturability significantly limit their application in compact mobile systems [9,10]. Composite BPs, which combine good corrosion resistance, high mechanical strength, and ease of molding, are regarded as ideal candidates and a promising future direction for BP development. Nevertheless, their practical application is currently hindered by challenges related to high production cost, non-uniform conductivity, and complex fabrication processes [11,12]. In comparison, metal BPs are more suitable for large-scale manufacturing owing to their low cost, low gas permeability, and satisfactory balance between electrical and thermal conductivity. However, a major drawback is their susceptibility to metal dissolution and surface passivation, both of which can deteriorate cell performance. Metal dissolution under cathodic operation potentials of PEMFCs (e.g., +0.6 V vs. SCE (saturated calomel electrode)) can lead to the degradation of the proton exchange membrane through the migration of dissolved metal ions, while surface passivation results in drastically increased interfacial contact resistance (ICR) due to the formation of semiconducting or insulating oxide films [13,14].

To address these issues, numerous studies have shown that utilizing suitable coatings can effectively improve the corrosion resistance and electrical conductivity of metal BPs [2]. Regrettably, limited research has been conducted on the durability of these coatings under actual production and operational conditions. Coating degradation is inevitable during long-term corrosion, which can result in localized corrosion of the underlying metal BPs near damaged coating areas. The corrosion resistance and service life of the coating are also closely related to the properties of the metallic substrate [15,16]. Meanwhile, recent advances have highlighted alloy design as a promising approach to mitigate the corrosion–ICR dilemma [17]. Our previous work revealed that NiTi alloy exhibits significantly decreased ICR values compared to commercial Ti BPs. In addition, the NiTi alloy showed fairly low current densities (1.28 µA cm^−2^) during 8-h potentiostatic tests, only slightly above the U.S. Department of Energy (DOE) standard (≤1 µA cm^−2^) [18]. H. Zhu et al. [19] investigated the effects of cold rolling, annealing time, solution temperature, and fluoride concentration in simulated PEMFC environments on the corrosion behavior of Ti-Nb-Ni foil, which consisted of β-Ti and NiTi_2_ phases. Their findings emphasized the beneficial role of traceable Nb, deposited via high-power impulse magnetron sputtering technology, in improving both electrical conductivity and corrosion resistance in PEMFC-like conditions. Furthermore, Ishikawa et al. [20] reported that the Nb_19_Ti_40_Ni_41_ alloy, featuring a cube-on-cube structure composed of β-Nb and B2-TiNi phases, exhibited high hydrogen permeability.

Inspired by these studies, this paper proposes a strategy to further improve the performance of NiTi alloy via Nb alloying in a simulated PEMFC environment. A novel NiTiNb alloy, comprising dispersed NiTi (B2) phase and continuous reticular β-Nb phase, was prepared by vacuum arc melting. The corrosion resistance and conductivity of the designed alloy were systematically investigated through electrochemical experiments, ICR measurements, and detailed characterization.

## 2. Results and Discussion

**Figure 1:**
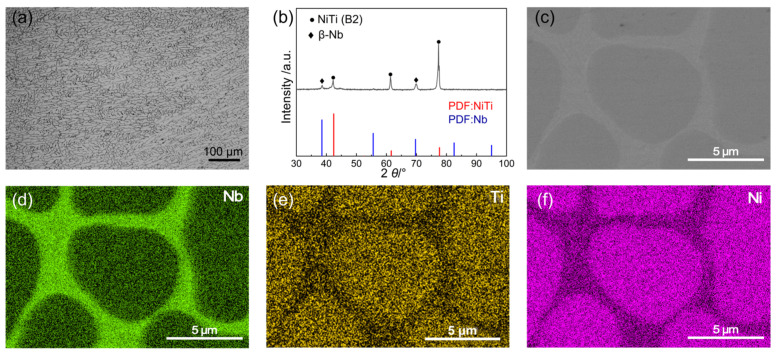
(**a**) Optical micrograph (OM), (**b**) X-ray diffraction (XRD) pattern, and (**c**) scanning electron microscopy (SEM) image of the NiTiNb alloy, along with the corresponding elemental distribution maps for (**d**) Nb, (**e**) Ti, and (**f**) Ni.

To explore the structural advantages of Nb-alloyed NiTi alloy, the phase composition and microstructure of the NiTiNb alloy, which are critical factors influencing its performance as BPs in PEMFCs, were systematically analyzed, as shown in Figure 1. The optical micrograph (OM) reveals a homogeneous as-cast microstructure characterized by fine, cellular-like dendrites (Figure 1a). This morphology closely resembles that of unalloyed NiTi, owing to the identical vacuum arc melting preparation method used for both alloys. During solidification, the relatively small ingot size (~20 mm) establishes a thermal gradient that promotes directional grain growth along the heat flow [21]. In comparison, pure Ti and pure Ni exhibit much coarser primary equiaxed grains, with a small number of twins observed in pure Ti (Figure S1 in Supplementary Materials). The X-ray diffraction (XRD) pattern (Figure 1b) confirms that the NiTiNb alloy mainly consists of the NiTi (B2) phase (JCPDS No. 96-901-4020) and the β-Nb phase (JCPDS No. 96-151-2525) [22,23] (both featuring a body-centered cubic (BCC) crystal structure), consistent with the phase composition typically reported in the literature for the Ni_47_Ti_44_Nb_9_ alloy [24]. The introduction of Nb leads to a eutectic-like microstructure, as evidenced by the scanning electron microscopy (SEM) image in Figure 1c and further supported by the corresponding energy dispersive spectroscopy (EDS) in Figure 1d–f. The β-Nb phase is continuously distributed along the grain boundaries, where a small amount of NiTi (B2) phase is also present, appearing as brighter areas in the SEM image. The grain interiors are predominantly composed of the NiTi (B2) matrix. Additionally, Nb atoms are partially substituted into the NiTi (B2) lattice. Given the larger atomic radius of Nb, this substitution induces lattice expansion, which manifests as a leftward shift of the diffraction peaks in the XRD pattern. Such a refined and homogeneous microstructure, along with the presence of a supersaturated solid solution and a continuous β-Nb phase network at grain boundaries, contributes to improved mechanical strength and enhanced workability of the NiTiNb alloy. These characteristics are particularly advantageous for BP applications in PEMFCs.

**Figure 2:**
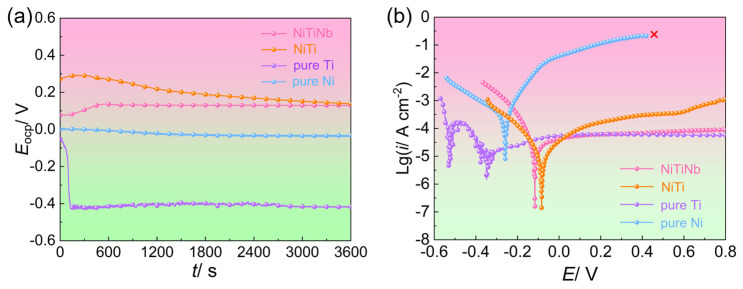
(**a**) Open circuit potential versus time (*E*_OCP_-t) curves and (**b**) potentiodynamic polarization curves of the NiTiNb alloy, NiTi alloy, pure Ti, and pure Ni.

The open circuit potential versus time (*E*_OCP_-t) curves and potentiodynamic polarization curves of the NiTiNb alloy, NiTi alloy, pure Ti, and pure Ni are shown in Figure 2, with key electrochemical parameters summarized in Table 1. Clearly, both the *E*_OCP_-t curves of NiTi alloy and pure Ti display fluctuations and downward drifts before stabilizing. This behavior can be attributed to localized dissolution of their native passive films during initial immersion in the halide-containing (e.g., F^−^) solution and the resulting instability during the dissolution–passivation process [25]. Pure Ni, on the other hand, exhibits a continuous decrease in open circuit potential (*E*_OCP_), implying persistent surface degradation and poor corrosion resistance. In comparison, the NiTiNb alloy demonstrates a brief initial rise in *E*_OCP_ followed by rapid stabilization into a smooth plateau, indicating the admirable stability of the formed passive films, which offers an effective barrier to improve corrosion resistance. All *E*_OCP_ values tend to stabilize after long-term immersion (3600 s). At the steady state, the *E*_OCP_ of NiTiNb alloy (0.13 V) is quite close to that of NiTi alloy (0.14 V). Both values are markedly nobler than those of pure Ni (−0.03 V) and pure Ti (−0.42 V), confirming the strong corrosion resistance tendency of NiTiNb alloy.

Potentiodynamic polarization curves shown in Figure 2b further support these observations. The relative order of corrosion potentials (*E*_corr_) among the four materials aligns well with their *E*_OCP_ values. Both NiTiNb and NiTi alloys exhibit similarly low corrosion current densities (*i*_corr_), approximately one order of magnitude lower than that of pure Ti and two orders lower than pure Ni. Notably, no passivation feature is observed in the anodic branch of pure Ni, with the anodic current density continuously rising until reaching the measurement limit (0.1 A) at 0.42 V. Pure Ti undergoes an unstable sequence involving initial passivation, transpassive dissolution, and re-passivation before reaching a quasi-stable state, accompanied by pronounced current fluctuations. The dissolution process can also be observed at the initial stage of immersion in the *E*_OCP_-t curve of pure Ti (Figure 2a). In contrast, anodic branches of NiTi and NiTiNb alloys change directly into the passive region without the active-to-passive transition, suggesting their superior passivation characteristics, as the passive films form rapidly and almost spontaneously at *E*_corr_ [26]. However, compared to ambient conditions [18], the presence of F^−^ and elevated temperature under air bubbling significantly reduce the stability of passive films on NiTi surfaces, leading to gradually increasing passive current density (*i*_pass_). Remarkably, the NiTiNb alloy maintains a consistently low *i*_pass_ under harsh conditions, a value quite close to that of pure Ti. This result highlights its excellent electrochemical stability and corrosion resistance, underscoring the strong potential of NiTiNb alloy as a durable and protective BP material for PEMFC applications.

**Figure 3:**
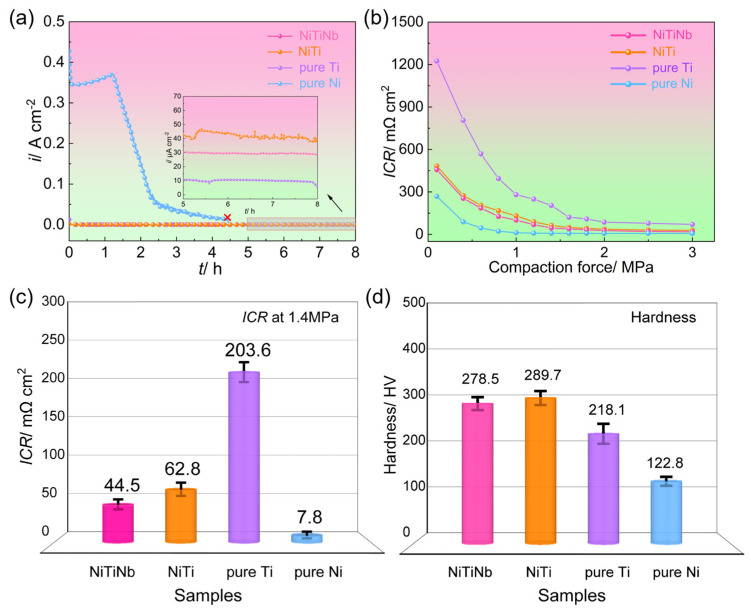
(**a**) Potentiostatic polarization curves, (**b**) interfacial contact resistance (ICR) values under varying compaction force and (**c**) 1.4 MPa, and (**d**) hardness of the NiTiNb alloy, NiTi alloy, pure Ti, and pure Ni.

Figure 3 presents the core properties of metal materials as BPs for PEMFC applications, including the corrosion durability, ICR, and mechanical hardness. The potentiostatic polarization curves, measured in a simulated PEMFC cathodic environment at 70 °C with air bubbling, reveal significant performance variations among the four tested materials. Pure Ni exhibits obviously higher cathodic current density (*i*_c_) than the other materials and turns into complete failure within 4.5 h due to severe dissolution. Pure Ti shows the lowest *i*_c_ value of 11.2 μA cm^−2^ but suffers from considerable electrochemical instability. The NiTiNb alloy emerges as the most promising candidate, demonstrating not only superior corrosion resistance (29.8 μA cm^−2^, representing a 26% improvement over the NiTi alloy) but also exceptional electrochemical stability. This advantage becomes particularly evident under ambient temperature and non-aerated conditions, where NiTiNb achieves a low current density of 0.90 μA cm^−2^ (Figure S2 in Supplementary Materials), significantly surpassing both the DOE target (<1 μA cm^−2^) and the NiTi alloy performance (1.28 μA cm^−2^) by 30% [18,27]. Importantly, even under accelerated corrosion conditions at elevated temperatures with aeration, the NiTiNb alloy consistently outperforms most uncoated metallic materials while maintaining the most stable electrochemical behavior across all test conditions [28,29,30,31].

Figure 3b,c illustrate the variations in ICR of the four tested materials as a function of compressive stress, with particular emphasis on 1.4 MPa, a typical assembly pressure in PEMFCs [32,33]. All materials show a characteristic decline in ICR as compressive stress increases, featuring an initial rapid decrease followed by gradual stabilization due to the enhanced effective contact area between the BP and the gas diffusion layer (GDL) [34]. The ICR ranking remains consistent across all pressure conditions: pure Ti > NiTi alloy > NiTiNb alloy > pure Ni. At 1.4 MPa, pure Ti demonstrates exceptionally high ICR (203.6 mΩ cm^2^), exceeding NiTi (62.8 mΩ cm^2^) and NiTiNb (44.5 mΩ cm^2^) by one order of magnitude and surpassing pure Ni (7.8 mΩ cm^2^) by two orders. This trend highlights the inherent trade-off between corrosion resistance and electrical conductivity in metallic BPs [7]. Pure Ti achieves the lowest *i*_c_ due to its pronounced passivation, but this comes at the cost of severely compromised conductivity. Conversely, pure Ni’s inability to form stable passive films results in poor corrosion resistance but excellent electrical contact. Our previous work has shown that NiTi intermetallic can effectively balance these two properties by combining the advantages of Ti and Ni [18]. The present results reveal that Nb alloying further optimizes this balance, with NiTiNb achieving both enhanced corrosion resistance and 29% lower ICR compared to NiTi.

Furthermore, the NiTiNb alloy exhibits comparable microhardness to the NiTi alloy while surpassing both pure Ti and pure Ni (Figure 3d), demonstrating excellent mechanical properties suitable for PEMFC BP applications. In summary, Nb alloying of NiTi alloy achieves simultaneous enhancement in both corrosion resistance and electrical conductivity while maintaining superior mechanical strength. These combined advantages position NiTiNb as a highly promising candidate material for metallic BP substrates in practical PEMFC applications.

**Figure 4:**
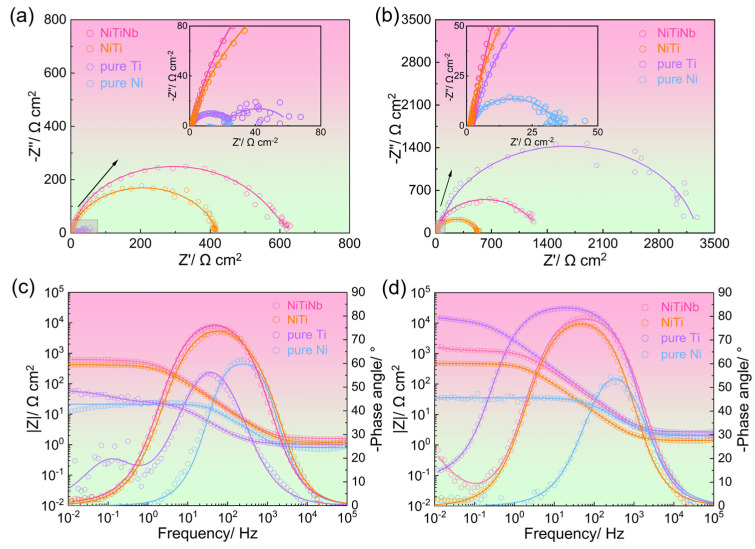
The electrochemical impedance spectroscopy (EIS) results for NiTiNb alloy, NiTi alloy, pure Ti, and pure Ni, presented as (**a**,**b**) Nyquist plots and (**c**,**d**) Bode-phase plots, measured (**a**,**c**) before and (**b**,**d**) after potentiostatic polarization.

Electrochemical impedance spectroscopy (EIS), SEM, and X-ray photoelectron spectroscopy (XPS) were employed to investigate the surface states of different BP materials in a simulated PEMFC environment, which are critical factors determining both corrosion resistance and electrical conductivity. Figure 4 exhibits the comparative EIS results obtained before and after potentiostatic polarization. As shown in Figure 4a,c, the impedance magnitude (|Z|) before potentiostatic polarization follows a clear descending order: NiTiNb alloy > NiTi alloy > pure Ti > pure Ni. This trend confirms the superior intrinsic corrosion resistance of NiTiNb alloy at the initial stage compared to other BP materials, consistent with the results of *i*_corr_ obtained from potentiodynamic polarization curves (Figure 2b). After potentiostatic polarization, pure Ti shows the highest |Z| (Figure 4b,d) due to the semiconductive passive film formation, which can offer corrosion resistance but simultaneously result in undesirably high ICR. The phase angle plots reveal distinct electrochemical responses (Figure 4c,d). Pure Ni shows only one single time constant at medium frequencies (~10^3^ Hz) both before and after potentiostatic polarization, indicating its metallic surface state is devoid of effective passivation, which explains its poor corrosion resistance and low ICR. In contrast, other BP materials exhibit an additional time constant at higher frequencies that merges with the metallic characteristic time constant, leading to relatively stable phase angles across the medium–low frequencies. Nevertheless, all tested materials display high and slanted impedance modulus values, confirming the presence of the protective passive films formed on the surfaces [26,35].

**Figure 5:**
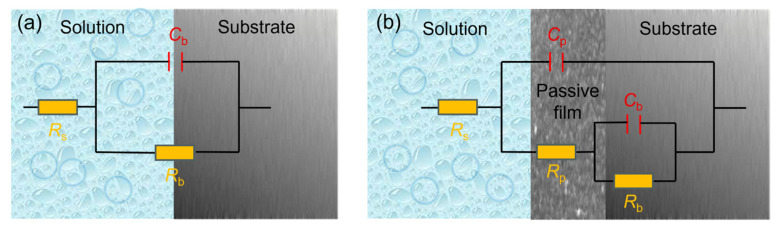
Equivalent electrical circuits used for EIS fitting: (**a**) one-time constant model; (**b**) two-time constant model.

Based on the characteristic description of EIS plots, the equivalent circuit proposed by A. K. Iversen [36] was used to fit the pure Ni data (Figure 5a), reflecting its charge transfer behavior at the metal/electrolyte interface without stable passivation. For other materials, the equivalent circuit from C. Boissy et al. [37] was applied (Figure 5b), accounting for both charge transfer and the capacitive response from the passive film/electrolyte interface. In these models, *R*_s_ represents the solution resistance. *C*_b_ and *R*_b_ correspond to the double-layer capacitance and charge transfer resistance, respectively. *C*_p_ and *R*_p_ denote the capacitance and resistance of the passive film. The “n” is the CPE exponent (0–1), reflecting deviation from ideal capacitance. The fitted parameters are summarized in Table 2, and the fitting lines are also shown in Figure 4. As shown in Table 2, the obtained chi-square (*χ*^2^) values are on the order of 10^−3^, indicating that the used equivalent circuits appropriately represent the actual electrochemical processes that occurred on the sample surfaces [38].

As quantified in Table 2, pure Ni consistently exhibits low *R*_b_ both before and after potentiostatic polarization, implying its poor corrosion resistance. In contrast, pure Ti shows a significant increase in *R*_b_ and *R*_p_ coupled with a remarkable decrease in *C*_b_ and *C*_p_ due to the formation of a semiconductive passive film barrier. It is worth noting that after potentiostatic polarization, the NiTiNb alloy shows a much higher *R*_p_ and lower *C*_p_ compared to the NiTi alloy, confirming enhancement of anti-corrosion performance. Importantly, while maintaining comparable *C*_p_ values to pure Ti, the NiTiNb alloy shows significantly smaller *R*_p_, demonstrating an optimal balance between protective barrier effects (enhancing corrosion resistance) and improved electrical conductivity (beneficial for ICR). These results demonstrate that the Nb alloying strategy successfully overcomes the typical trade-off between corrosion protection and electrical conductivity in metal BP materials.

**Figure 6:**
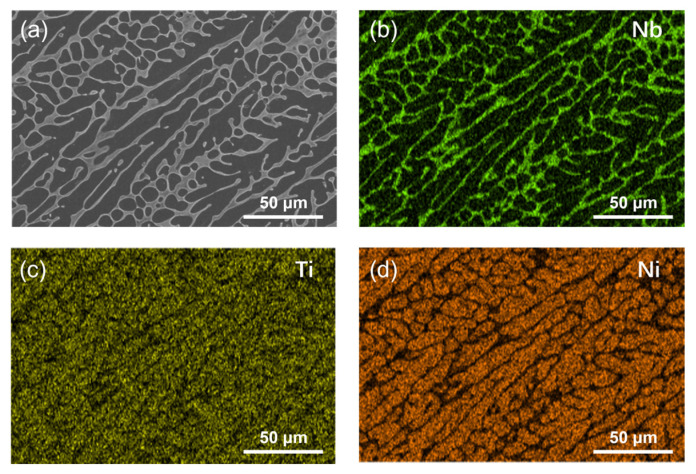
SEM image of (**a**) NiTiNb alloy after potentiostatic polarization and (**b**–**d**) corresponding elemental distribution maps.

Figure 6 presents the SEM image and corresponding elemental mapping of NiTiNb alloy after potentiostatic polarization. The surface reveals remarkable corrosion resistance as evidenced by the absence of visible corrosion damage and the preservation of its original as-cast cellular-like dendrite microstructure. Notably, EDS analysis demonstrates distinct Nb enrichment along the continuous intergranular network. Complementary observations from Figure S3 in Supplementary Materials show that NiTi alloy develops initial pitting corrosion under more aggressive heated and aerated conditions, while pure Ti maintains its corrosion-free surface. In stark contrast, pure Ni suffers from severe intergranular corrosion damage. These morphological characteristics correlate well with the electrochemical analysis results.

**Figure 7:**
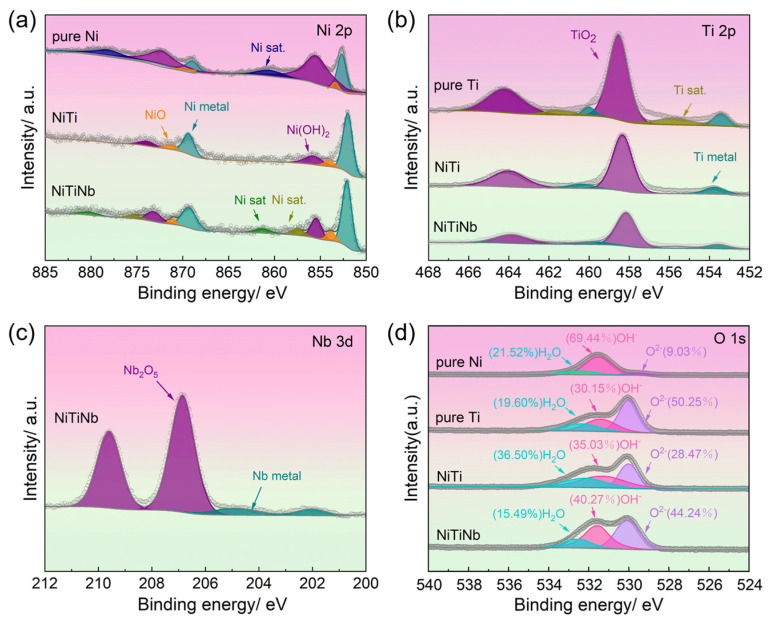
X-ray photoelectron spectroscopy (XPS) analysis results of NiTiNb alloy, NiTi alloy, pure Ti, and pure Ni: (**a**) Ni 2p, (**b**) Ti 2p, (**c**) Nb 3d, (**d**) O 1s.

Figure 7 presents the XPS spectra from the surfaces of NiTiNb alloy, NiTi alloy, pure Ti, and pure Ni after potentiostatic polarization, with detailed peak deconvolution revealing distinct surface chemical states. The Ni 2p spectrum (Figure 7a) exhibits three characteristic components, namely Ni(OH)_2_ at 855.6 eV, NiO at 853.7 eV, and metallic Ni at 852.6 eV, while the Ti 2p spectrum (Figure 7b) shows TiO_2_ at 458.5 eV and metallic Ti at 454.1 eV. Additionally, the Nb 3d spectrum of the NiTiNb alloy (Figure 7c) demonstrates the coexistence of Nb_2_O_5_ (207.1 eV) and metallic Nb (202.4 eV). Notably, the O 1s spectrum (Figure 7d) reveals three major contributions, namely lattice oxygen (O^2−^ at 530.0 eV), hydroxyl groups (OH^−^ at 531.6 eV), and absorbed water (H_2_O at 532.3 eV), with O^2−^/OH^−^ serving as a critical indicator of passive film quality. The presence of bound water facilitates the capture of dissolved metal cations on the electrode surface, thereby promoting the development of denser passivation film to mitigate corrosion damage [25]. Quantitative analysis of surface chemical species for all materials is provided in Table 3.

XPS spectral analysis reveals fundamental differences in surface chemical states that govern the performance of these BP materials. Pure Ni surfaces are dominated by Ni(OH)_2_ formation, exhibiting a remarkably low O^2−^/OH^−^ ratio of 0.13 that reflects severe corrosion and ineffective passivation. In contrast, pure Ti develops a TiO_2_-rich passive film with a high O^2−^/OH^−^ ratio of 1.67, suggesting excellent corrosion resistance at the expense of electrical conductivity. The NiTiNb alloy demonstrates an optimal compromise with a moderate O^2−^/OH^−^ ratio of 1.1 (versus 0.81 for NiTi), maintaining 76.11% metallic Ni content (compared to 71.94% in NiTi). This high metallic content implies the presence of a thin yet protective oxide layer, further supported by a TiO_2_-Nb_2_O_5_ composite passive film. This unique surface composition explains the material’s superior performance. On the one hand, the TiO_2_-Nb_2_O_5_ composite passive film provides corrosion resistance comparable to pure Ti. On the other hand, the continuous Nb/Nb_2_O_5_ network along grain boundaries establishes conductive pathways, effectively reducing ICR. These results are consistent with previous findings demonstrating that Nb/Nb_2_O_5_ coatings significantly improve both corrosion resistance and electrical conductivity in 316L stainless steel as well as Ti-based BPs [39], with similar enhancements reported for Nb_2_O_5_-modified Ti-6Al-4V alloys [40]. As a result, the present study demonstrates that bulk Nb alloying in NiTi achieves comparable benefits through in situ formation of a multifunctional surface structure during polarization, effectively overcoming the conventional trade-off between corrosion resistance and conductivity in metallic BP materials.

## 3. Materials and Methods

### 3.1. Materials

In this study, a NiTiNb alloy with a nominal atomic ratio of Ni:Ti:Nb = 47:44:9, representative of typical commercial compositions, was selected as the primary research material. For comparison, a binary NiTi alloy with a nominal atomic ratio of Ni:Ti = 51:49, along with pure Ti and pure Ni, which were previously studied as PEMFC bipolar plate (BP) materials under ambient conditions, were also included [18]. Both NiTiNb and NiTi alloys were fabricated using the vacuum arc melting process (WK-I, Physcience Opto-electronics Co., Ltd., Beijing, China). To ensure compositional homogeneity, small ingots (~10 mm in diameter) were first remelted six times with flipping between melts. These ingots were then consolidated and subjected to three additional remelting cycles, also with flipping, to form the final ingots (~25 mm in diameter). High-purity raw materials were used, including electrolytic nickel (purity > 99.9%), titanium sponge (purity > 99.7%), and niobium particle (purity > 99.9%). Pure Ti and pure Ni samples were purchased from Shengshida Metal Materials Co. Ltd., Xingtai, China. All materials were cut into thin plates with dimensions of 20 mm × 10 mm × 1 mm using wire electrical discharge machining. The sample surfaces were mechanically ground using progressively finer silicon carbide papers up to 2000 grit, polished with a 1 μm diamond suspension, and sequentially cleaned in anhydrous ethanol and deionized water under ultrasonic agitation. These prepared samples were used for subsequent electrochemical tests, microstructural characterization, and phase identification.

### 3.2. Electrochemical Measurements

Electrochemical measurements were carried out using a CHI660E electrochemical workstation (Chenhua, Shanghai, China) in a conventional three-electrode cell. The cell configuration was composed of a saturated calomel electrode (SCE) as the reference, a platinum electrode as the counter, and the prepared sample as the working electrode. All measured potentials (V_SCE_) were converted to values relative to the standard hydrogen electrode (SHE) using the following relationship: V_SHE_ = V_SCE_ + 0.244 V.

Prior to each test, the working electrode was stabilized under the open circuit condition for 3600 s to establish a steady open circuit potential (*E*_OCP_). Potentiodynamic polarization measurements were conducted by scanning the potential from *E*_OCP_ − 0.25 V to *E*_OCP_ + 1.00 V at a rate of 1 mV/s. Potentiostatic polarization was carried out at a simulated cathodic potential of 0.85 V_SHE_ for 8 h [41]. Electrochemical impedance spectroscopy (EIS) was performed at the *E*_OCP_ over a frequency range from 10 mHz to 100 kHz with a sinusoidal perturbation of 10 mV [42]. All electrochemical tests were conducted in a simulated PEMFC environment, consisting of 0.5 M H_2_SO_4_ containing 2 ppm F^−^ at 70 °C, with continuous air bubbling [43]. Each test was repeated at least three times under consistent conditions to ensure parameter suitability and data accuracy.

### 3.3. ICR Measurements

**Figure 8:**
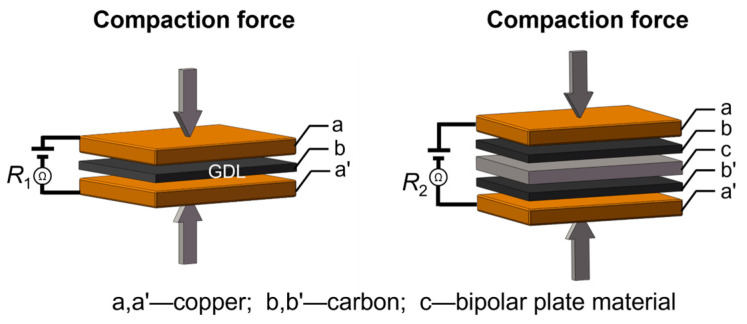
The schematic diagram for testing the *R*_1_ and *R*_2_ values for calculating the ICR value.

Interfacial contact resistance (ICR) measurements were conducted after potentiostatic polarization tests, following the method proposed by Wang et al. [44], using a DC low-resistance meter (TH2516B, Tonghui, Changzhou, China). The ICR was calculated using Equation (1):

(1) $$ICR=(R_{2}-R_{1})/2$$

where *R*_2_ is the measured total resistance, *R*_1_ is the resistance attributed to the gas diffusion layer (GDL) with carbon paper, and *A*_c_ is the contact area between the sample and the carbon paper. The procedure for determining *R*_1_ and *R*_2_ is illustrated in the schematic diagram shown in Figure 8. Compaction pressures ranging from 0.1 to 3.0 MPa were applied, and the corresponding ICR value under each pressure was obtained by averaging three separate measurements taken at different locations on the sample surface to minimize local variability.

### 3.4. Characterization

The microstructure of the alloys was characterized using optical microscopy (OM, DM2700 M, Leica, Wetzlar, Germany) and field-emission scanning electron microscopy (FE-SEM, Apreo S Hivac, Thermo Fisher, Waltham, MA, USA). Prior to OM observation, the samples were etched with a mixed solution of HF:HNO_3_:H_2_O in a volume ratio of 1:4:5 to reveal the microstructure. Elemental distributions were analyzed by energy dispersive spectroscopy (EDS, Ultim Live 100X, Oxford, Abingdon, UK) integrated with FE-SEM. Phase identification was performed using X-ray diffraction (XRD, SmartLab SE, Rigaku, Tokyo, Japan) with CuKα radiation. The chemical compositions and elemental valence states of the passive film formed on the sample surfaces were analyzed by X-ray photoelectron spectroscopy (XPS, AXIS SUPRA+, Shimadzu, Kyoto, Japan) equipped with an AlKα X-ray source (1486.6 eV, probing depth < 10 nm).

## 4. Conclusions

This study systematically evaluates the performance of NiTiNb alloy as a potential BP material in simulated PEMFC environments (70 °C, 0.5 M H_2_SO_4_ + 2 ppm F^−^ with air bubbling). Results reveal that Nb alloying maintains the intrinsic balance between corrosion protection and electrical conductivity of NiTi alloy while significantly enhancing both properties. After 8 h of polarization, the NiTiNb alloy demonstrates a 26% current density reduction and a 29% ICR decrease compared to the NiTi alloy, effectively overcoming the conventional performance trade-off in metallic BPs. The alloy also displays superior electrochemical stability and significantly enhanced microhardness relative to pure Ti and Ni. These exceptional properties originate from the alloy’s unique dual-phase microstructure, featuring an NiTi (B2) matrix with a continuous β-Nb network along grain boundaries. The in situ formation of TiO_2_-Nb_2_O_5_ composite passive film enhances corrosion resistance, while the interconnected Nb/Nb_2_O_5_ network and preserved metallic Ni domains synergistically improve electrical conductivity. These findings establish NiTiNb as a highly promising BP substrate for PEMFC and provide an effective optimization strategy through compositional modification.

## Figures and Tables

**Table 1:** The electrochemical parameters extracted from the *E*_OCP_-t curves and the potentiodynamic polarization curves.

Samples	*E*_OCP_ at Steady State (V)	*E*_OCP_ Standard Deviation During 2400–3600 s (mV)	*E*_corr_ (V)	*i*_corr_ (μA cm^−2^)
NiTiNb	0.13	0.21	−0.12	5.01
NiTi	0.14	8.98	−0.08	6.51
Pure Ti	−0.42	4.76	−0.53	75.95
Pure Ni	−0.03	1.43	−0.26	221.23

**Table 2:** Electrochemical parameters extracted from equivalent circuit fitting of EIS data.

Before	**Elements**	***R*_s_** **(Ω cm^2^)**	***C*_b_** **(Ω^−1^ cm^−2^ s^n^)**	***n*_1_**	***R*_b_** **(Ω cm^2^)**	***C*_p_** **(Ω^−1^ cm^−2^ s^n^)**	***n*_2_**	***R*_p_** **(Ω cm^2^)**	**∑*χ*^2^**
	NiTiNb	1.59	7.02 × 10^−3^	0.53	137.6	1.88 × 10^−4^	0.91	528.2	5.34 × 10^−3^
	NiTi	1.20	7.99 × 10^−5^	0.88	7.66	2.05 × 10^−4^	0.77	413.7	8.67 × 10^−3^
	Pure Ti	1.03	6.23 × 10^−2^	0.82	32.11	1.82 × 10^−3^	0.85	24.97	9.62 × 10^−3^
	Pure Ni	0.79	2.68 × 10^−4^	0.94	20.7	-	-	-	7.24 × 10^−3^
After	**Elements**	***R*_s_** **(Ω cm^2^)**	***C*_b_** **(Ω^−1^ cm^−2^ s^n^)**	***n*_1_**	***R*_b_** **(Ω cm^2^)**	***C*_p_** **(Ω^−1^ cm^−2^ s^n^)**	***n*_2_**	***R*_p_** **(Ω cm^2^)**	**∑*χ*^2^**
	NiTiNb	1.97	1.18 × 10^−3^	0.58	376.7	8.26 × 10^−5^	0.94	1030	3.18 × 10^−3^
	NiTi	1.38	1.02 × 10^−4^	0.93	363.6	1.49 × 10^−4^	0.80	520.2	6.55 × 10^−3^
	Pure Ti	1.71	2.05 × 10^−4^	0.89	346.8	9.81 × 10^−5^	0.99	2935	9.28 × 10^−3^
	Pure Ni	1.67	1.60 × 10^−4^	0.89	32.9	-	-	-	9.08 × 10^−3^

**Table 3:** The estimated ratios of different chemical states at the surface of the NiTiNb alloy, NiTi alloy, pure Ti, and pure Ni.

Samples	Ni 2p (%)		Ti 2p (%)		Nb 3d (%)		O^2−^/OH^−^
	NiO + Ni(OH)_2_	Metallic Ni	TiO_2_	Metallic Ti	Nb_2_O_5_	Metallic Nb	
NiTiNb	23.87	76.11	91.37	8.63	92.53	7.47	1.10
NiTi	28.06	71.94	90.89	9.11	/	/	0.81
Pure Ti	/	/	82.31	17.69	/	/	1.67
Pure Ni	87.65	12.35	/	/	/	/	0.13

## Data Availability

The data presented in this study are available on request from the corresponding author.

## Supplementary Materials

The following supporting information can be downloaded at https://www.mdpi.com/article/10.3390/molecules30173658/s1, Figure S1. Optical micrographs of (a) NiTi alloy, (b) pure Ti, and (c) pure Ni; Figure S2. Potentiostatic polarization curve of the NiTiNb alloy tested under ambient conditions with no air bubbling; Figure S3. Scanning electron microscopy (SEM) images of (a) NiTi alloy, (b) pure Ti, and (c) pure Ni after potentiostatic polarization.
