# Supplementary material for: Improving Anti-Corrosion and Conductivity of NiTi Alloy Bipolar Plate Used for PEMFCs via Nb Alloying
Source: Molecules. 2025 Sep 8;30(17):3658. doi: 10.3390/molecules30173658 (PMC12429972; doi:10.3390/molecules30173658)
Supplement: Supplementary file 1 [file molecules-30-03658-s001.zip › molecules-3836394-supplementary.pdf]

Supplementary Materials for

Improving Anti-Corrosion and Conductivity of NiTi alloy Bipolar Plate used for PEMFCs via Nb alloying

Ziyang Niu [1,†], Yingping Li [1,†], Yuanyuan Li [1,2,*], Xiaofen Wang [3,4], Yumin Pan [3], Zhuo He [3], Guohong Zhang [2], Zhen Wang [2] and Qiongyu Zhou [3,*]

1 The State Key Laboratory of Refractories and Metallurgy, Wuhan University of Science and Technology, Wuhan 430081, China; niuziyang@wust.edu.cn (Z.N.); liyingping@wust.edu.cn (Y.L.)
2 Analytical and Testing Center, Wuhan University of Science and Technology, Wuhan 430081, China; zhangguohong@wust.edu.cn (G.Z.); wangzhen@wust.edu.cn (Z.W.)
3 Key Laboratory of Green Surface Technology and Functional Coatings for Materials, China National Light Industry, Foshan University, Foshan 528000, China; wangxiaofen@shu.edu.cn (X.W.); 20230580130@stu.fosu.edu.cn (Y.P.); 20220580212@stu.fosu.edu.cn (Z.H.)
4 School of Materials Science and Engineering, Shanghai University, Shanghai 200444, China
* Correspondence: liyuanyuan@wust.edu.cn (Y.L.); zhouzhouqiongyu@fosu.edu.cn (Q.Z.). Tel: +86-135-12741496 (ext. 430081) (Y.L.); +86-159-70962829 (ext. 528000) (Q.Z.)
† These authors contributed equally to this work.

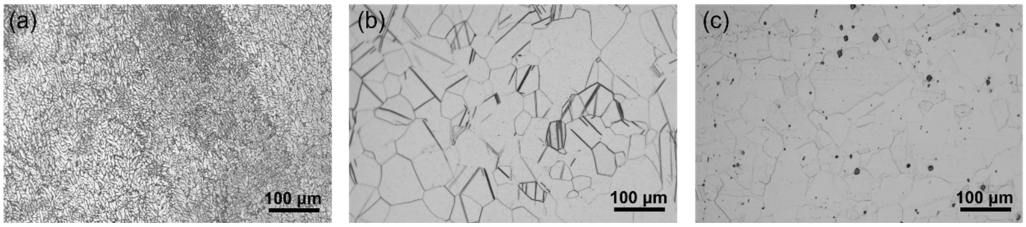

Figure S1. Optical micrographs of (a) NiTi alloy, (b) pure Ti and (c) pure Ni.

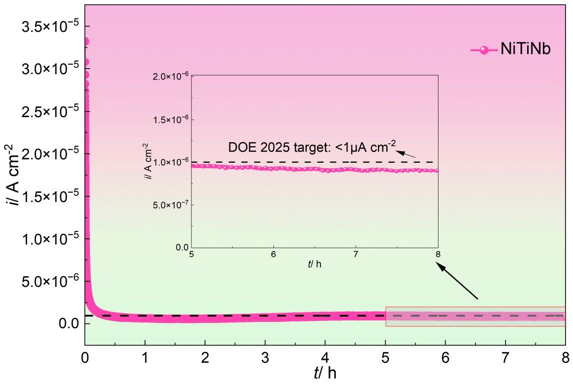

Figure S2. Potentiostatic polarization curve of the NiTiNb alloy tested under ambient condition with no air bubbling.

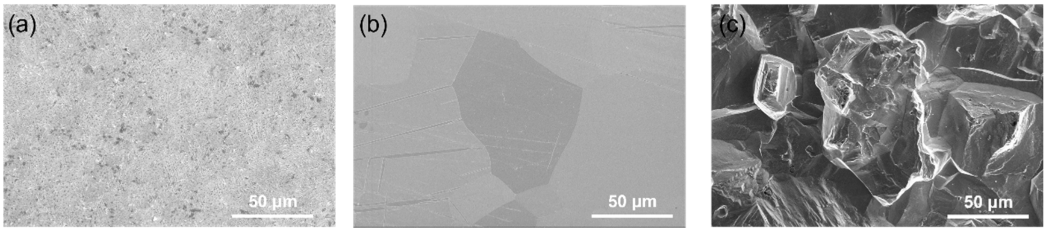

Figure S3. Scanning electron microscopy **(**SEM) images of (a) NiTi alloy, (b) pure Ti and (c) pure Ni after potentiostatic polarization.
